# Ferroptosis, a New Insight Into Acute Lung Injury

**DOI:** 10.3389/fphar.2021.709538

**Published:** 2021-08-02

**Authors:** Xiaofang Yin, Guisong Zhu, Qian Wang, Yuan Dong Fu, Juan Wang, Biao Xu

**Affiliations:** ^1^ Intensive Care Uint, Nanjing Hospital of Chinese Medicine Affiliated to Nanjing University of Chinese Medicine, Nanjing, China; ^2^ Department of Respiration, Jiangsu Province Hospital of Chinese Medicine, Affiliated Hospital of Nanjing University of Chinese Medicine, Nanjing, China

**Keywords:** ferroptosis, acute lung injury, iron metabolism, lipid peroxidation, ferrostatin-1, lipoxstatin-1, iASPP

## Abstract

Acute lung injury (ALI), a common and critical illness with high morbidity and mortality, is caused by multiple causes. It has been confirmed that oxidative stress plays an important role in the development of ALI. Ferroptosis, a newly discovered programmed cell death in 2012, is characterized by iron-dependent lipid peroxidation and involved in many diseases. To date, compelling evidence reveals the emerging role of ferroptosis in the pathophysiological process of ALI. Here, we review the role of ferroptosis in the pathogenesis of ALI and its therapeutic potential in ALI.

## Introduction

**FIGURE 1:**
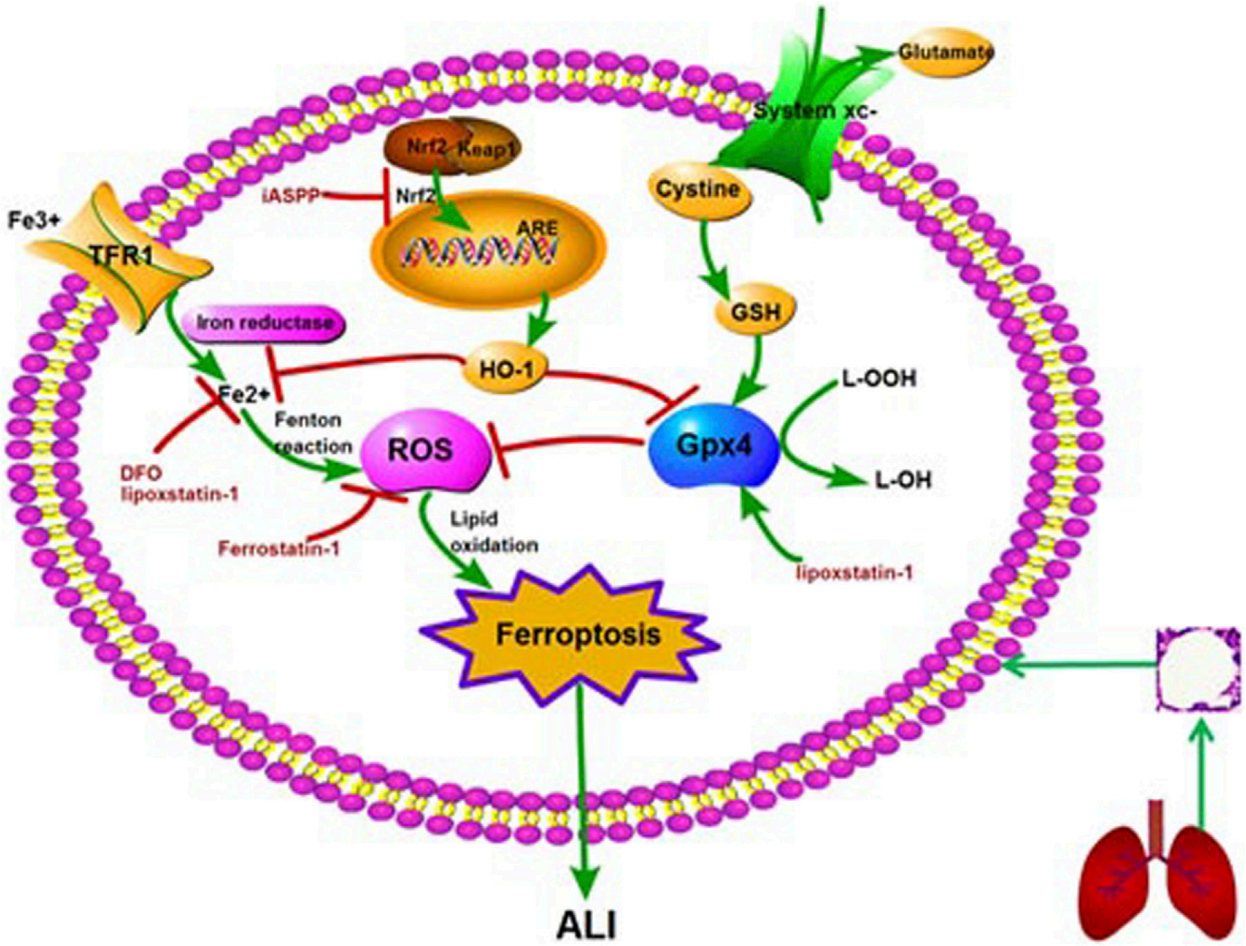
The molecular mechanism and regulation of ferroptosis in ALI. Note: ALI: acute lung injury; GSH: glutathione peroxidase 4; ROS: reactive oxygen species; Nrf2: nuclear factor erythroid 2-related factor 2; HO-1: heme oxygenase-1; ARE: antioxidant response element; TFR1: transferrin receptor 1; DFO: deferoxamine; iASSP: inhibitor of apoptosis-stimulating protein of p53.

Acute lung injury (ALI), a common and critical illness with high morbidity and mortality, is caused by a variety of factors, including pulmonary and extrapulmonary factors (Ware et al., 2000; Mutlu et al., 2006; Matthay et al., 2019). The pathogenesis of ALI is not fully understood, and there is still no effective targeted intervention. Therefore, it is of great significance to study the pathogenesis and treatment of ALI. The pathogenesis of ALI was previously believed to involve inflammation, coagulation, oxidative stress, repair and so on (Matthay et al., 2019). In 2012, Dixon et al. proposed ferroptosis, a new concept of cell death. Studies have shown that ferroptosis is closely related to tumor, nervous system disease, infection, ischemia/reperfusion (I/R) injury, kidney injury and other diseases (Friedmann et al., 2014; Linkermann et al., 2014; Stockwell et al., 2017; Dar et al., 2018; Amaral et al., 2019; Hu et al., 2019; Mou et al., 2019; Han et al., 2020; Mao et al., 2020). In recent years, ferroptosis has also been confirmed to contribute to lipopolysaccharide (LPS)-induced ALI, intestinal I/R-induced ALI, oleic acid-induced ALI, and acute radiation-induced lung injury (RILI) (Li et al., 2019; Liu et al., 2020; Dong et al., 2020; Li et al., 2020; Zhou et al., 2019). In addition, ferroptosis inhibitors ferrostatin-1 and lipoxstatin-1, as well as inhibitor of apoptosis-stimulating protein of p53 (iASPP), can mediate protective effects against ALI by inhibiting ferroptosis (Liu et al., 2020; Li et al., 2020). This review summarizes the research progress, regulatory mechanism and therapeutic potential of ferroptosis in ALI (Figure 1).

### Ferroptosis Is Iron-dependent Lipid Peroxidation

Ferroptosis was first discovered as a unique form of cell death by Dixon et al. when studying the mechanism of erastin killing tumor cells with RAS mutation in 2012 (Dixon et al., 2012). As a programmed cell death, ferroptosis is quite different from apoptosis and autophagy in morphology (Table 1). The characteristics of ferroptosis include decreased mitochondrial crista, increased mitochondrial membrane density, along with ruptured mitochondrial outer membrane as observed under electron microscopy, but the integrity of nucleus remains (Kazan et al., 2019). Besides mitochondria, other organelles such as golgi, endoplasmic reticulum and lysosome, are also involved in ferroptosis. Golgi stress-related lipid peroxidation, endoplasmic reticulum-related oxidative stress, and lysosome dysfunction can induce ferroptosis (Wu et al., 2020). The biological properties of ferroptosis are characterized by a large amount of iron accumulation and membrane lipid peroxidation products in the process of cell death. At present, the mechanism of ferroptosis mainly focuses on oxidative damage and iron metabolism (Stockwell et al., 2017; Li et al., 2020). Lipid peroxidation and iron metabolism signaling are recognized as the central mediators of ferroptosis (Xie et al., 2016).

**TABLE 1:** Morphological comparison between ferroptosis, apoptosis, necrosis, pyroptosis and autophagy (Kazan et al., 2019); (Dixon et al., 2012); (Wu et al., 2020); (Stockwell et al., 2017); (Li et al., 2020).

Cell death	Ferroptosis	Apoptosis	Necrosis	Pyroptosis	Autophagy
Morphological characteristics	Shrinkage of mitochondria, reduction of mitochondrial cristae, increase of mitochondrial density, rupture of mitochondrial outer membrane	Blebbing of plasma membrane, reduction in cellular and nuclear volume, nuclear rupture	Plasma membrane swelling, organelle swelling, moderate chromatin condensation	Karyopyknosis, cell edema, rupture of cell membrane	Formation of double-membrane autophagic lysosomes
Biochemical characteristics	Iron accumulation and lipid peroxidation, inhibition of system Xc-, depletion of GSH, and inhibition of GPX4	Caspases activation, fragmentation of oligonucleotide DNA	RIP1-RIP3 complex formation, activation of effector protein MLKL, necrotic body formation (complex IIb)	Caspase-1 mediated, assembly of inflammasome, release of inflammatory factors	The activity of lysosome increasing, digestion and degradation of many enzymes

Cystine/glutamate transporter (system Xc-) is an important antioxidant, composed of two subunits, SLC7A11 and SLC3A2L. It can transfer cystine into cells and excrete glutamate out of cells. Cystine and glutamate are exchanged by system Xc-in a ratio of 1:1. Cystine is reduced to cysteine by system Xc- and participates in the synthesis of glutathione (GSH) (Chen et al., 2015). Then GSH is reduced to corresponding alcohols under the action of glutathione peroxidase 4 (Gpx4) (Bridges et al., 2012). Therefore, inhibition of system Xc- and Gpx4 can reduce Cystine uptake and GSH synthesis, leading to oxidative damage and even cell death. This process is also different from apoptosis and autophagy.

As a substrate for the synthesis of lipid signal mediators, the amount and location of polyunsaturated fatty acids (PUFA) determine the degree of lipid peroxidation in cells. The ferroptosis signal transmitted by PUFA depends on the esterification of membrane-forming phospholipids and oxidation (D'Herde et al., 2017). Acyl CoA synthase long chain family member 4 (ACSL4) and Lysophosphatidylcholine acyltransferase 3 (LPCAT3) are involved in the biosynthesis and remodeling of polyunsaturated fatty acid PE (PUFA-PE) in cell membrane. With the activation of ACSL4, the free PUFA could be esterified with the help of LPCAT3, and then bound to the membrane phospholipid to form PUFA-PE. Therefore, the up-regulation of ACSL4 is considered as a biomarker and contributor of ferroptosis. PUFA-PE can promote lipoxygenase (LOXs)-mediated enzymatic reaction to form lipid hydroperoxides. Therefore, the depletion of LOXs in cells can prevent ferroptosis induced by erastin (Yang et al., 2016).

Circulating iron exists in the form of ferric iron (Fe^3+^) by binding to transferrin. Fe^3+^ iron is imported into the cell via the membrane protein transferrin receptor 1 (TFR1) and then locates in the endosome. Then the Fe^3+^ iron is reduced to ferrous iron (Fe^2+^) by reductase in the endosome. The release of Fe^2+^ can be mediated by divalent metal transporter1 (DMT1) from the endosome into the cytoplasm that is a labile iron pool. Ferritin is an iron storage protein complex, where excessive iron is stored in. It includes ferritin light chain (FTL) and ferritin heavy chain1 (FTH1) (Harrison et al., 1996). FTH1 catalyzes the conversion of Fe^2+^ form into the Fe^3+^ form and then the Fe^3+^ iron is bound to the ferritin shell, thus reducing the level of free iron. Excessive iron can lead to ferroptosis by producing ROS through Fenton reaction (Wang et al., 2018).

### Key Regulators of Ferroptosis

#### Gpx4

There are eight subtypes of glutathione peroxidase (Gpx) in mammals, among which Gpx4 is the key regulator of ferroptosis (Brigelius-Flohe et al., 2013; Conrad et al., 2015). Gpx4, a single copy gene located on chromosome 19, was isolated and purified from pig liver by Ursini and colleagues in 1982 (Forcina et al., 2019; Ursini et al., 1982; Hirschhorn et al., 2019). It is a selenoprotein that can repair oxidative damage of lipid cells. Gpx4 is unique among Gpx subtypes as it is the only enzyme capable of reducing the esterified oxidized fatty acids and cholesterol hydroperoxides (Conrad et al., 2015). Gpx4 can convert intracellular toxic lipid hydrogen peroxide (L-OOH) into nontoxic lipid alcohol (L-OH), and promote the decomposition of hydrogen peroxide (H2O2), which can protect cell membrane from oxidative damage (Forcina et al., 2019). Therefore, Gpx4 is essential for preventing cell damage and maintaining tissue homeostasis (Wortmann et al., 2013). It was found that a large number of ferroptosis occurred in renal tubular cells when Gpx4 gene was knocked out (Friedmann et al., 2014). Specific blockade of Gpx4 can lead to destruction of muscles, neurons, and other cells, suggesting that Gpx4 is essential for survival of adult cells. In conclusion, inactivation of Gpx4 can lead to accumulation of lipid peroxide and ferroptosis, and Gpx4 plays a negative regulatory role in the process of ferroptosis (Forcina et al., 2019; Kinowaki et al., 2018).

#### System Xc−

System Xc− is an important intracellular antioxidant system. It is an amino acid transporter expressed on mammalian cell membrane, composed of two subunits, SLC7A11 and SLC3A2L. Intracellular glutamate is exchanged with extracellular cystine via system Xc− by the ratio of 1:1. Cystine is involved in the synthesis of GSH, an important intracellular free radical scavenger. Inhibition of system Xc− can lead to a rapid decrease in intracellular GSH level and rapid ferroptosis (Bridges et al., 2012; Dixon et al., 2012). Studies have also found that tumor suppressor p53 can inhibit the uptake of cystine by inhibiting SLC7A11, thus inducing ferroptosis (Jiang et al., 2015).

#### Nrf2

Nuclear factor erythroid 2-related factor 2 (Nrf2) is an important transcription factor regulating cellular oxidative stress response, and antioxidant response element (ARE) is a downstream signal molecule of Nrf2(Canning et al., 2015; Xie et al., 2016; Krajka-Kuźniak et al., 2017). After activation of Nrf2/ARE signaling pathway, a classical signaling pathway, a series of cell protective genes can be induced, such as heme oxygenase−1 (HO−1), nicotinamide adenine dinucleotide phosphate quinone oxidoreductase (NQO1), glutathione peroxidase (GSH−Px) and so on (Chen et al., 2018). Nrf2 plays a very important role in ferroptosis by regulating iron homeostasis and lipid peroxidation (Sun et al., 2016; Kerins et al., 2018; Dodson et al., 2019). Studies show that Nrf2/HO-1 signaling pathway can regulate anti-inflammatory, antioxidant stress, and ferroptosis, which plays a multi-organ protective role (Zhang et al., 2019; Jiang et al., 2020). Nrf2-keap1 pathway can change tumor microenvironment and affect tumor growth by up regulating xCT (SLC7A11 or system Xc−). Nrf2 overexpression or Keap1 knockout can inhibit ferroptosis, accelerate the proliferation of glioma cells, and reduce the survival rate of tumor patients, suggesting a potential target for tumor treatment (Sartori et al., 2010).

### Ferroptosis and ALI

The clinical manifestations of ALI are characterized by diffuse pulmonary infiltration, refractory hypoxemia and respiratory distress. The pathological manifestations are injury of pulmonary capillary endothelial cells and alveolar epithelial cells, and diffuse alveolar and interstitial edema. As is well known, ALI can be caused by various extrapulmonary factors (such as sepsis, surgery, burns, fluid resuscitation, severe pancreatitis,etc.) and pulmonary factors (such as pulmonary inflammation, pulmonary contusion, aspiration,etc.). However, the confirmed pathogenesis of ALI is very complex, mainly involving the uncontrolled inflammatory reaction, the regulation of aquaporin, the imbalance of coagulation/fibrinolysis system, apoptosis, autophagy, pyrosis and so on (Sartori et al., 2010; Kovarova et al., 2012; Matthay et al., 2019).

Under normal circumstances, the lung relies on the phagocytosis of macrophages, transferrin in secretion, antioxidant molecules on the surface of respiratory tract epithelium, and respiratory ciliary expectoration system to maintain iron homeostasis. Once the protective mechanisms are destroyed by endogenous or exogenous factors, oxidative stress injury occurs in the lung (Turi et al., 2004). It was found that there was iron excess in the lower respiratory tract of ALI patients (Stites et al., 1999; Ghio et al., 2003). Iron accumulation can lead to inflammatory reaction, oxidative stress and mitochondrial dysfunction, and eventually cause lung damage through ferroptosis (Yoshida et al., 2019). Iron excess can also induce or aggravate hyperoxia-induced lung injury in patients with mechanical ventilation or diving operators, while intravenous deferoxamine can attenuate lung injury (Sha et al., 2019). Iron also plays an important role in hypoxia-induced lung injury. Iron supplementation can promote inflammatory response and oxidative stress, and aggravates lung injury induced by high altitude in rats (Salama et al., 2014). In addition, it has been found that the total GSH decreased and oxidized glutathione (GSSG) increased in alveolar epithelial lining fluid of ALI patients and animal models (Schmidt et al., 2004; Britt et al., 2014). In conclusion, in the pathological process of ALI, the release of various reactive oxygen species and the generation of free radicals can damage alveolar epithelial cells, and iron overload can further promote the conversion of hydrogen peroxide into free radicals through Fenton reaction, which increases the cytotoxicity, thus promoting the occurrence and development of ALI (Zhang et al., 2019). In conclusion, these studies suggest that iron metabolism and oxidative stress may involve in the pathogenesisof ALI. With the development of ferroptosis research, more and more studies have found that ferroptosis is involved in the pathogenesis of ALI. It has been confirmed that ferroptosis exists in some animal models or cell models of ALI.

### Ferroptosis Involved in ALI

#### Ferroptosis in Sepsis-Induced Lung Injury

Sepsis is a systemic inflammatory response syndrome caused by severe infection, with rapid progression and poor prognosis. ALI often occurs in the early stage of sepsis, but no effective treatments are currently available for it (Rubenfeld et al., 2005). Sepsis-induced lung injury is essentially an acute pathological injury of lung tissue caused by uncontrolled inflammatory reaction. *In vitro* (Liu et al., 2020), it was found that the expression of ferroptosis markers, SLC7A11, and GPx4, were down-regulated, while the levels of malondialdehyde (MDA) and total iron were significantly increased in a dose-dependent manner after lipopolysaccharide (LPS) intervention on the human bronchial epithelial cell line, BEAS-2B. Ferrostatin-1, an inhibitor of ferroptosis, could reverse the above effects, suggesting that ferroptosis played a very important role in the pathogenesis of LPS-induced ALI. *In vivo* (Yu et al., 2014; Liu et al., 2020), similar conclusions were drawn in ALI models established by intratracheal or intravenous injection of LPS. Ferroptosis may participate in LPS-induced ALI through Nrf2/ARE signaling pathway (Yu et al., 2014; Liu et al., 2020). Another study showed that HO−1 played a protective role in the pathogenesis of sepsis-induced lung injury, and artesunate could improve sepsis-induced lung injury by activating Nrf2 and promoting HO−1 expression (Luo et al., 2014; Cao et al., 2016; see Table 2). In conclusion, ferroptosis may be a potential therapeutic target for sepsis-induced lung injury, thus indicating the therapeutic potential of ferroptosis inhibitors for it.

**TABLE 2:** Ferroptosis in several types of ALI.

		Types of ALI	Manifestation	Signal pathway
Ferroptosis		Sepsis-induced lung injury	Down-regulation of SLC7A11 and GPx4, MDA and total iron increasing Yu et al. (2014); Yu et al. (2014); Liu et al. (2020); Liu et al. (2020)	Nrf2/ARE
		Intestinal I/R-Induced ALI	Pulmonary edema, lipid peroxidation Dong et al. (2020); Meng et al. (2016); Li et al. (2020)	Nrf2/HIF-1α/TF
		Oleic acid-induced ALI	Iron overload, decrease in glutathione, GPx4 and ferritin, increase in MDA content Lee et al. (2014); Zhou et al. (2019)	Unclear
		Radiation-induced lung injury	A large amount of ROS producing Li et al. (2019)	Unclear

#### Ferroptosis in Intestinal I/R-Induced ALI

Intestinal I/R injury can be caused by severe trauma, extensive burns, severe infection, shock, intestinal obstruction, cardiac surgery, etc. Once intestinal I/R injury occurs, intestinal mucosal barrier is destroyed, intestinal bacteria and toxins are translocated, and then a large number of cytokines and inflammatory mediators are released into blood circulation, leading to systemic inflammatory response and injury of distant organs. The lung is the earliest and most vulnerable organ, known as intestinal I/R-induced ALI, which plays a vital role in the development of multiple organ dysfunction syndrome (MODS) (de Perrot et al., 2003; Marco et al., 2007; Mörs et al., 2017; Li et al., 2020). So far, the pathogenesis of intestinal I/R-induced ALI has not been fully elucidated, and there is no specific medicine.

In the past, apoptosis was considered to be the main regulatory cell death mode in various ischemic injury models. However, in recent years, more and more studies have found that ferroptosis is the main driving factor of ischemic injury (Tonnus et al., 2017). Studies *in vivo* and *in vitro* have confirmed that ferroptosis occurs in type II alveolar epithelial cells of mice with intestinal I/R injury, and ferroptosis is involved in intestinal I/R-induced ALI (Dong et al., 2020; Li et al., 2020). Ferroptosis inhibitor ferrostatin-1 can improve intestinal I/R-induced ALI by alleviating pulmonary edema and inhibiting lipid peroxidation, while iron can reverse the above effects. It has been found that Nrf2 regulates ferroptosis by promoting the expression of HO−1 and SLC7A11, which plays a protective role in ferroptosis. Nrf2 may be a key regulator of intestinal I/R-induced ALI (Meng et al., 2016; Dong et al., 2020). Further research shows that iASPP can alleviate intestinal I/R-induced ALI and ferroptosis through Nrf2/HIF-1α/TF signaling pathway (Li et al., 2020; see Table 2). At present, the mechanism research is still in its infancy.

#### Ferroptosis in Oleic Acid-Induced ALI

Intravenous injection of oleic acid is one of the methods to establish ALI models (Zhou et al., 2014). Once oleic acid microbubbles enter pulmonary capillaries, it results in pulmonary vascular congestion, increased capillary permeability and pulmonary interstitial edema, which is consistent with the pathological changes of ALI (Lee et al., 2014). In mice injected with oleic acid, iron overload, decrease in glutathione levels, GPx4 (a marker of ferroptosis) and ferritin, and increase in MDA content, were observed in lung tissues, accompanied with morphological changes of ferroptosis such as mitochondrial wrinkle and mitochondrial membrane rupture (Zhou et al., 2019; see Table 2). However, the specific molecular signaling pathways remain unclear.

#### Ferroptosis in RILI

The incidence of RILI was 16.7–50.3%, which increased the mortality and disability of lung cancer patients (S N et al., 2019). Oxidative damage of lung tissue, induced by a large amount of Reactive Oxygen Species (ROS) produced by radiation, is a key factor in the pathogenesis of RILI(Li et al., 2019). It has been found that ferroptosis played an important role in RILI, and ferroptosis inhibitor significantly reduced ROS in lung tissue and inflammatory factors in serum (Li et al., 2019; see Table 2).

### Treatment of ALI by Targeting Ferroptosis

Ferroptosis is characterized by a large amount of iron accumulation and lipid peroxidation. Accordingly, the therapeutic targets for ferroptosis should focus on inhibiting iron metabolism and lipid peroxidation. Iron metabolism inhibitors and iron chelators, such as deferoxamine (DFO), can inhibit ferroptosis by inhibiting iron uptake (Wang et al., 2020). Inhibitors of lipid metabolism inhibit polyunsaturated fatty acids (PUFA) incorporation into phospholipid membranes, such as thiazolidinediones and knockdown of long-chain acyl-CoA synthetases (ACSL4) (Dixon et al., 2015; Doll et al., 2017). Ferroptosis inhibitor ferrostatin-1 can improve intestinal I/R-induced ALI by inhibiting lipid peroxidation and alleviating pulmonary edema (Dong et al., 2020). It can also alleviate sepsis-induced ALI by promoting the expression of SLC7A11 and GPx4 and reducing the levels of MDA and iron in lung tissue (Liu et al., 2020). Another ferroptosis inhibitor, lipoxstatin-1, can promote the expression of GSH and GPx4, and reduce the content of MDA, iron and transferrin. In addition, lipoxstatin-1 can reverse the effect of erastin on promoting ferroptosis, and alleviate intestinal I/R-induced ALI, suggesting the therapeutic potential of lipoxstatin-1 (Li et al., 2020). IASPP, which is a known inhibitor of p53 transcriptional activity, mainly exists in the cytoplasm. IASPP inhibits ferroptosis through Nrf2/HIF-1α/TF signaling pathway and plays a protective role in intestinal I/R-induced ALI, as demonstrated in MLE-2 cells (Li et al., 2020). Although these ferroptosis inhibitors have been proved to have the effect of improving ALI, they are still in animal models and/or *in vitro* studies, lack of clinical evidence (see Table 3).

**TABLE 3:** Impact of ferroptosis inhibitors on ALI.

Reagent	Target	Impact on ALI
Ferrostatin-1	Lipid peroxidation Dong et al. (2020) GPX4, SLC7A11 Liu et al. (2020)	Alleviating sepsis-induced ALI, improving intestinal I/R-induced ALI
Panaxydol	Keap1-Nrf2/HO−1 pathway Li et al. (2021)	Alleviating LPS-induced ALI
Lipoxstatin-1	GSH and GPX4, Nrf2 pathway Li et al. (2020)	Alleviating intestinal I/R-induced ALI
IASPP	Nrf2/HIF−1α/TF Li et al. (2020)	Protecting against intestinal I/R-induced ALI
Artesunate	HO−1Cao et al. (2016); Luo et al. (2014)	Protecting against sepsis-induced lung injury
Sevoflurane	HO−1 Liu et al. (2021)	Protecting against LPS-induced ALI

## Conclusion and Perspectives

As a new form of programmed cell death, ferroptosis is known to mainly involve in tumor, nervous system diseases, I/R injury and acute kidney injury. In recent years, more and more studies have found that ferroptosis is also involved in the pathogenesis of ALI. Ferroptosis has been confirmed in many ALI animal models or cell models, but its specific mechanism has not been fully elucidated. Ferroptosis may be a target for the treatment of ALI, focusing on the inhibition of iron metabolism and lipid peroxidation. Ferrostatin-1, lipoxstatin-1 and iASPP have been proved to have the effects on inhibiting ferroptosis and protecting ALI. However, they only stay in animal models and/or *in vitro* studies, lack of clinical evidence. In the future, more comprehensive and in-depth scientific researches are needed to further explore the relationship between ferroptosis and ALI, to provide more theoretical basis for clinical work.
